# Supplementary material for: From sequence to enzyme mechanism using multi-label machine learning
Source: BMC Bioinformatics. 2014 May 19;15:150. doi: 10.1186/1471-2105-15-150 (PMC4229970; doi:10.1186/1471-2105-15-150)
Supplement: Additional file 2 — Java code of ml2db. Additional file ml2db_code.tar.gz contains the Java source code to run the multi-label machine learning experiments and save the results to database. The code’s Javadoc is included. [file 1471-2105-15-150-S2.zip › additional file 2/ml2db/utils/doc/edu/cornell/lassp/houle/RngPack/RandomShuffle.html]

RandomShuffle


JavaScript is disabled on your browser.


- Overview
- Package
- Class
- Use
- Tree
- Deprecated
- Index
- Help

- Prev Class
- Next Class

- Frames
- No Frames

- All Classes

- Summary:
- Nested |
- Field |
- Constr |
- Method

- Detail:
- Field |
- Constr |
- Method


edu.cornell.lassp.houle.RngPack

## Class RandomShuffle

- java.lang.Object
- - cern.colt.PersistentObject
  - - edu.cornell.lassp.houle.RngPack.RandomElement
    - - edu.cornell.lassp.houle.RngPack.RandomShuffle

- All Implemented Interfaces:
  :   java.io.Serializable, java.lang.Cloneable

  ---

    

  ```
  public class RandomShuffle
  extends RandomElement
  ```

  RandomShuffle uses one random number generator to shuffle the numbers
  produced by another to obliterate sequential correlations. To initialize a
  RandomShuffle, pass it two RandomElements. The first RandomElement is used to
  generate a table of random numbers and the second is used to choose one from
  the table. An example of usage is,

  ```
   RandomElement markov=new RandomShuffle(new Ranecu(),new Ranmar(),32)
  ```

  which would generate a deck of 32 numbers from RANECU and use
  RANMAR to choose from the deck.
    
  **References:**
  - F. James; Comp. Phys. Comm. **60** (1990) p
    329-344- D. Knuth; The Art of Computer Programming vol. 2, sec 3.2.2

  Source code  is available.

  Version:
  :   1.0

  Author:
  :   Paul Houle  (E-mail: houle@msc.cornell.edu)

  See Also:
  :   Serialized Form

- - ### Constructor Summary

    Constructors

    Constructor and Description

    `RandomShuffle(RandomElement ga,
    RandomElement gb,
    int ds)`
  - ### Method Summary

    Methods

    | Modifier and Type | Method and Description |
    | --- | --- |
    | `java.lang.Object` | `clone()` Returns a copy of the receiver. |
    | `double` | `raw()` The generator. |

    - ### Methods inherited from class edu.cornell.lassp.houle.RngPack.RandomElement

      `choose, choose, gaussian, gaussian, powlaw, raw, raw, uniform`
    - ### Methods inherited from class java.lang.Object

      `equals, getClass, hashCode, notify, notifyAll, toString, wait, wait, wait`

- - ### Constructor Detail


    - #### RandomShuffle

      ```
      public RandomShuffle(RandomElement ga,
                   RandomElement gb,
                   int ds)
      ```

      Parameters:
      :   `ga` - generator to fill shuffle deck
      :   `gb` - geberator to choose from shuffle deck
      :   `ds` - the size of the shuffle deck
  - ### Method Detail


    - #### clone

      ```
      public java.lang.Object clone()
      ```

      Returns a copy of the receiver.

      **Overrides:**
      :   `clone` in class `PersistentObject`

      Returns:
      :   a copy of the receiver.


    - #### raw

      ```
      public double raw()
      ```

      The generator.

      **Specified by:**
      :   `raw` in class `RandomElement`

      Returns:
      :   a random double in the range (0,1).

      See Also:
      :   `RandomElement.raw()`


- Overview
- Package
- Class
- Use
- Tree
- Deprecated
- Index
- Help

- Prev Class
- Next Class

- Frames
- No Frames

- All Classes

- Summary:
- Nested |
- Field |
- Constr |
- Method

- Detail:
- Field |
- Constr |
- Method
